# Distribution modelling for Neotropical freshwater stingrays *Potamotrygon brachyura* and *Potamotrygon motoro* (Myliobatiformes, Potamotrygonidae) in the Uruguay River basin

**DOI:** 10.1111/jfb.70219

**Published:** 2025-09-11

**Authors:** Danilo Araujo Soares Pereira, Roberto E. Reis, Nelson F. Fontoura

**Affiliations:** ^1^ Laboratory of Ichthyology, Science and Technology Museum Pontifical Catholic University of Rio Grande do Sul (PUCRS) Porto Alegre Brazil

**Keywords:** biogeography, Elasmobranchii, fisheries, La Plata basin, logistic regression, South America

## Abstract

This study aimed to identify geographical distribution patterns of the giant short‐tailed river stingray *Potamotrygon brachyura* and the motoro stingray *Potamotrygon motoro* in the Uruguay River basin. Data on presence/absence of stingrays were based on fishers' knowledge accessed by interviews through expeditions in Brazil, Argentina and Uruguay. The significance in independent variables (environmental descriptors) of elevation (*p* = 0.00 for both species), upstream distance (*p* = 0.02 for *P. brachyura* and 0.03 for *P. motoro*) and downstream distance (*p* = 0.00 for both species) explained the presence of stingrays in the main water bodies of lower Uruguay and their absence throughout upper Uruguay more than the biogeographical barrier of the Salto de Yucumã (*p* = 0.99 for both species) and basin area (*p* = 0.42 for *P. brachyura* and *p* = 0.43 for *P. motoro*) in the last steps. The construction of logistic models also provided high sensitivity (96.3%–97.5% for *P. brachyura* and 62.3%–71.7% for *P. motoro*) and specificity (86.8% for *P. brachyura* and 85.4%–86.5% for *P. motoro*) results between observed and presumed distribution for both species, with values of false positive and false negative varying between 1.3%–14.1% and 6.4%–9.2%, respectively. Further studies are still necessary not only in the Uruguay River basin or the La Plata basin complex, but for all South America, considering potential changes in the state of knowledge of freshwater biodiversity and its geographical distribution, including the possibility of undescribed species.

## 1 INTRODUCTION

The La Plata basin, also referred to as the Paraná‐Paraguay River system, stands as one of the largest drainages, most socioeconomically important and biodiverse basin complex in the Neotropical region and in Earth. This extensive basin encompasses the territories of Argentina, Bolivia, Brazil, Paraguay and Uruguay, with a total catchment area of 3,182,064 km^2^. Its natural resources configure a substantial role for biodiversity and human water security extending beyond its geographical boundaries (CIC/OAS, 2017). Within its different aquatic environments, including marine, brackish and particularly freshwater ecosystems, the La Plata basin complex ichthyofauna includes more than 1200 described species (Reis et al. 2016; Pereira, *pers. obs*.). This remarkable fish biodiversity sustains continuous new species descriptions every year (Fricke et al., 2024), with endemic species rates reaching up to half of the total fauna (Reis et al., 2016). Serving as habitat for large migratory fish and threatened megafauna species, including important fisheries resources (Barradas et al., 2012), this system includes a crucial biological corridor in the Southern Cone of South America: the Uruguay River.

**FIGURE 1:**
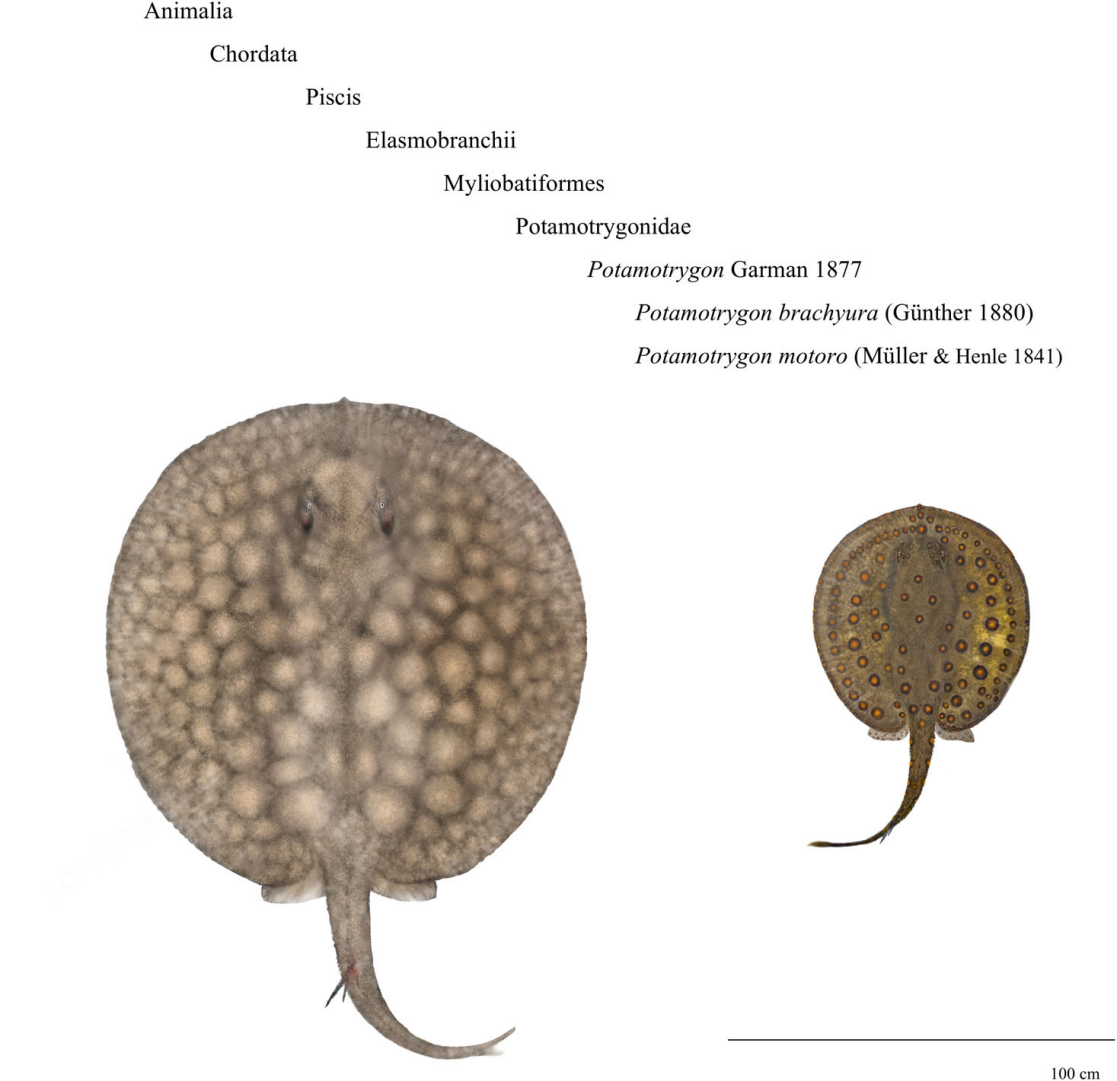
Neotropical freshwater stingrays (Potamotrygonidae) investigated in Uruguay River basin (La Plata basin, South America): The giant short‐tailed river stingray *Potamotrygon brachyura* (Günther 1880) (left) and the motoro freshwater stingray *Potamotrygon motoro* (Müller & Henle 1841) (right). Illustration: Danilo Araujo Soares Pereira.

**FIGURE 2:**
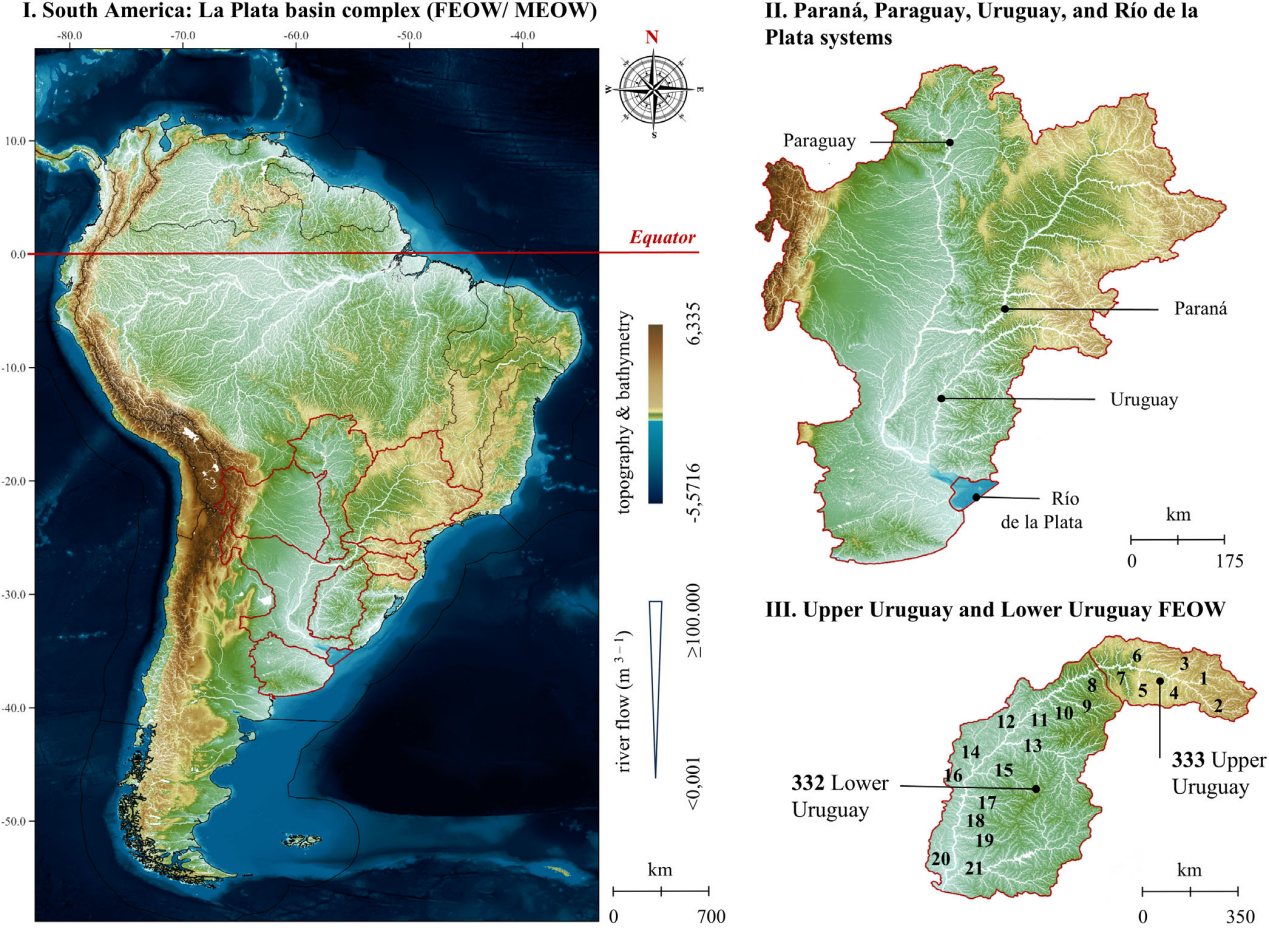
Map of the ecoregions of the La Plata and Uruguay basins. I. Biogeographical divisions of the La Plata basin and adjacent areas of South America, according to Freshwater Ecoregions of the World (FEOW) (Abell et al., 2008) proposed by Reis et al. (2016) and Marine Ecoregions of the World (MEOW) (Spalding et al., 2007). II. Identification of La Plata basin. III. Main water courses in the drainages of the Uruguay River: 1. Rio Canoas, 2. Rio Pelotas, 3. Rio do Peixe, 4. Rio Apuê e Forquilha, 5. Rio Passo Fundo, 6. Rio Chapecó, 7. Rio da Várzea, 8. Rio Buricá, 9. Rio Ijuí, 10. Rio Santa Rosa, 11. Rio Icamaquã, 12. Río Aguapey, 13. Rio Ibicuí, 14. Río Miriñay, 15. Rio Quaraí, 16. Río Mocoretá, 17. Río Arapey, 18. Río Dayman, 19. Río Queguay, 20. Río Gualeguaychú and 21. Río Negro. Bathymetric and topographic data obtained from global databases of the General Bathymetric Chart of the Oceans (GBECO, 2023), and river flow in logarithmic scale from HydroSheeds (Lehner & Grill, 2013). Maps: Danilo Araujo Soares Pereira.

The Uruguay River basin extends through the territories of Brazil, Argentina and Uruguay with an area of approximately 353,451 km^2^ or 11.1% of the entire La Plata basin (CIC/OAS, 2017). The Uruguay River has a length of 2200 km with altitude exceeding 1800 m (Figure 2) and an average annual discharge of 5725 m^3^ s^−1^. Its hydrographic region in Brazil alone covers an area of 174,199 km^2^ (CIC/OAS, 2017) with 405 municipalities and over 6 million inhabitants in the states of Rio Grande do Sul and Santa Catarina, supporting important agro‐industrial activities, hydroelectric power generation, urban water supply (ANA, 2015) and other activities – such as inland fishing (Barradas et al., 2012). According to data from the Brazilian National Water Agency (ANA, 2015), the Uruguay River basin presents critical contexts, including conflicts of interest over water use, historical deficiencies in basic sanitation and sewage treatment, and one of the highest energy generation potentials, with hydroelectric dams frequently related to problems involving floods and droughts (ANA, 2015; CIC/OAS, 2017). In addition to these human impacts on the environment, aspects such hydrological regimes, river connectivity, ecoregions interactions, habitat heterogeneity, latitudinal transitions and elevation gradients represent biogeographical determinants for the fish biodiversity and distribution in this basin (Abell et al., 2008; Barradas et al., 2012).

Although the Uruguay River basin is represented by iconic large‐sized species, such as the dourado *Salminus brasiliensis* (Cuvier 1816) and the spotted sorubim *Pseudoplatystoma corruscans* (Spix & Agassiz 1829), it is highlighted by the presence of two other rather unusual megafauna species: the freshwater stingrays (He et al., 2019). The short‐tailed giant stingray *Potamotrygon brachyura* (Günther 1880), the largest exclusively freshwater stingray on the planet, and the spotted or simply ‘motoro’ stingray *Potamotrygon motoro* (Müller & Henle 1841), the elasmobranch with the largest continental neotropical distribution. The family Potamotrygonidae (Myliobatiformes) is endemic to South America and includes all freshwater stingray species on the continent: the only Elasmobranchii completely adapted to the freshwater environment (Amazonas et al., 2024; Fontenelle et al., 2021; Lovejoy, 1996). In the last two decades, 20 new species of freshwater stingrays have been identified, representing exactly half of all the diversity formally recorded in the family Potamotrygonidae (FishBase, 2024; Fricke et al., 2024). In addition to 14 species of *Potamotrygon*, 5 new species of other genera have been described in the past 20 years, including a new genus, *Heliotrygon* (see Carvalho & Lovejoy, 2011; Loboda et al., 2021). Of the newly discovered species, only three do not belong to *Potamotrygon* Garman 1877, which is probably the most important taxonomic group among sharks and rays destined for aquariums and oceanariums around the world. In Brazil, there are records of at least 25 species, with 13 endemic species (see Silva & Loboda, 2019; Loboda et al., 2021 and the CAS in Fricke et al., 2024) and, possibly, at least 9 undescribed species in scientific collections (Ramos, 2017). In addition, as a fishery resource, these animals exhibit reproductive strategies with slow intrinsic population growth rates (Duncan et al., 2010; Ramos, 2017), and have been compared by some authors to sharks, skates, rays, stingrays and other marine elasmobranchs as k‐strategists (Charvet‐Almeida et al., 2005). Although there is regular trade for some species highly valued in the ornamental fish market, there are serious problems related to the lack of basic information about these animals and the existence of a parallel or even unidentified market in catches directed at subsistence, fish marketing and sport or amateur fishing (Ramos, 2017).

When this fishery resource is valued, pressures involve sophisticated strategies to contour regional barriers imposed by legislation involving the absence of data on its distribution (Lasso et al., 2016; Ramos, 2017). This lack of information gives rise to illegal trade based on false taxonomic identifications and natural occurrence (Charvet‐Almeida et al., 2005; Ramos, 2017). A striking example is the case of *Potamotrygon wallacei* Carvalho et al., 2016 (endemic to the Amazon basin), which for more than a decade was officially and legally traded as *Potamotrygon histrix* (Müller & Henle 1839) (endemic to the La Plata basin) until its formal description (Carvalho et al., 2016). These difficulties led the Brazilian Ministry of the Environment in cooperation with other countries to include the entire Brazilian populations of *Potamotrygon* spp. in Appendix III of the Convention on International Trade in Endangered Species of Wild Fauna and Flora (CITES), which lists species that can only be traded under regulation and cooperation between different parties (CITES, 2016; Ramos, 2017; Lasso et al., 2016). On the contrary, it is known that specimens of the group are used as capture fisheries resources in different regions without major controls or statistics (Lucifora et al., 2015).

Among freshwater elasmobranchs, the giant stingray (*P. brachyura*) can reach over 200 kg and is exploited by international fisheries for different purposes, including commercial, sportive and subsistence fishing (Ramos, 2017). *P. motoro* stands out as one of the most important species for the international aquarium fish market, having been the most captured and exported species in Brazil (Ramos, 2017), with serious gaps regarding its geographical distribution, wide genetic variation, multiple colour patterns and extremely high final sale values, sometimes even higher than USD 2000 per unit (Ramos, 2017). South American scientific committees for freshwater stingray request investigations on geographical distribution of both species as priority among urgent measures (Lasso et al., 2016). Other authorities such as Brazilian Ministry of Environment and CITES also confirm these taxa among species of major concern (Ramos, 2017), both occurring in the Uruguay River basin with pending details about their distributions (Bertaco et al., 2016; Lasso et al., 2016; Lucifora et al., 2015; Oddone et al., 2012; Serra et al., 2019; Sverlij et al., 1998).

Information derived from fishers' knowledge and the ecosystem approach to fisheries in Latin America (FAO, 2015) allows for examining species presence or absence as a dependent variable, intersecting with various environmental descriptors influencing biota distribution (Barradas et al., 2012). Lucifora et al. (2015) further contributed significant findings on the geographical distribution and conservation considerations of the giant stingray within the Paraná, Paraguay, Uruguay and Río de La Plata systems. By employing logistic models, these descriptors can be used as independent variables to statistically predict the geographical distribution of large species using observed and estimated data within the Uruguay River basin (Barradas et al., 2012). Consequently, this study aims to develop probabilistic distribution models for freshwater stingrays, specifically *P. brachyura* and *P. motoro* (Figure 1), based on occurrence records and key environmental descriptors across the global freshwater ecoregions of the Uruguay River basin (Figure 2).

## 2 MATERIALS AND METHODS

### 2.1 Study area

The La Plata basin is one of the largest river drainages in the world and the second largest in South America, following the Amazon basin. In hydrogeographical terms, the Paraná, Paraguay, Uruguay and Río de La Plata rivers drain areas of 1,510,513, 1,120,154, 353,451 and 190,113 km^2^, respectively (CIC/OAS, 2017). Its observed values of average annual flow were obtained for the Paraná River of 17,200 m^3^ s^−1^ (Corrientes, ARG), Paraguay of 3550 m^3^ s^−1^ (Porto Pilcomayo, Argentina and Asunción, Paraguay) and Uruguay of 4200 m^3^ s^−1^ (Paso de Los Libres, Argentina and Uruguaiana, Brazil) and estimated for about 27,000 m^3^ s^−1^ to the Río de La Plata itself (Argentina and Uruguay). The topographic and bathymetric variations in the basin range from 6200 m (Andes) to reach values at sea level (La Plata River estuary). In biogeographical terms, the La Plata basin is divided into eight Freshwater Ecoregions of the World (FEOW, Abell et al., 2008) and one Marine Ecoregion (MEOW, Spalding et al., 2007): 332. Lower Uruguay (LU, 270,190 km^2^, 8.23% of the total area constituted by aquatic ecoregions of La Plata basin added together): 333; upper Uruguay (UU, 78,848 km^2^, 2.40%): 342; Chaco (CH, 588,397 km^2^, 17.9%): 343; Paraguay (PY, 544,398 km^2^, 16.6%): 344; upper Paraná (UP, 825,108 km^2^, 25.1%): 345; lower Paraná (LP, 642,391 km^2^, 19.6%): 346; Iguaçu (IG, 67,408 km^2^, 2.05%): 347; Buenairense (BN, 265,042 km^2^, 8.08%): 182; and Río de la Plata (The detailed treatment of the La Plata Basin is entirely necessary, as it is constituted by the Uruguay River. The study area incorporates mathematical models and several discussions concerning neighbouring drainages within the La Plata Basin.) (Figure 2). Its freshwater fish biodiversity was previously estimated between 908 (CIC/OAS, 2017) and 924 species (Reis et al., 2016), increasing to 1107 native species expected in freshwater and 86 exclusively brackish and marine, including 7 species of freshwater stingrays exclusively, in addition to 35 non‐native fish introduced from other aquatic ecoregions (Araujo & Fontoura, 2024). Considering demographic and socioeconomic relevance, the land cover of the basin is configured in 58.4% of natural cover (or 12.9% of South America land cover), 41.0% agriculture (48.0% of South America land cover) and 0.54% urban (0.25% of South America land cover), covering 70% of all combined gross domestic product (GDP) of its five countries and a population of around 111 million inhabitants.

In terms of Uruguay River hydrogeographical conception, the main channel has an extension of approximately 2200 km (ANA, 2015). It is formed by the confluence of the Pelotas and Canoas rivers on the border between the Brazilian states of Santa Catarina and Rio Grande do Sul, acting as a geopolitical limit between Argentina and Brazil until the confluence of the Quaraí River, and then dividing Argentina and Uruguay along its length until its mouth in the Río de la Plata estuary. The Uruguay River basin is located in a temperate climate region, with average annual rainfall of 1624 mm distributed throughout the year, with the highest concentration of rainfall in the winter. Some authors cite regions of the Uruguay River as having the greatest thermal amplitude in Brazil, with temperatures ranging from −10 to 41°C, relative humidity ranging from 12% to 100% and well‐defined seasons (Querol et al., 2018). The altitude varies from sea level (Río de la Plata estuary) to values around 1800 m (Serra Geral) (GEBCO, 2023). According to biogeographical approaches, the upper Uruguay aquatic ecoregion (Abell et al., 2008) has a biodiversity of at least 235 fish species, whereas the lower Uruguay ecoregion has a specific richness of at least 295 species (Araujo & Fontoura, 2024). Among them, two distinct freshwater stingray species confirmed to be *P. brachyura* and *P. motoro* (Oddone et al., 2012; Bertaco et al., 2016; Lasso et al., 2016; Loureiro et al., 2023), although a third species is observed in some references without further support, such as the striped stingray *P. histrix* (Müller & Henle 1839) (Fricke et al., 2024).

From a demographic and socioeconomic point of view, land cover in the upper Uruguay River is divided between natural, agricultural and urban areas, with rates of 48.9%, 50.8% and 0.32%, respectively, whereas the lower Uruguay River occupies 57.1%, 42.7% and 0.18%, respectively (Araujo & Fontoura, 2024; Kobayashi et al., 2017). The Uruguay River basins or ecoregions have great social and economic relevance with transnational implications, especially due to their importance for public water supply, agro‐industrial activities and their hydroelectric potential (ANA, 2016; CIC/OAS, 2017). According to different parties involved in the Global Dam Watch (GDW) initiative, at least 292 dams are planned or become operative according to the Future Hydropower Reservoirs and Dams Database (FHRED) (Mulligan et al., 2021), in addition to about 2000 dams (irrigation reservoirs in general) mainly concentrated in the lower Uruguay River on the western border of the state of Rio Grande do Sul (Mulligan et al., 2020) alongside paddy fields and pastures (ANA, 2016; CIC/OAS, 2017).

## 3 DATA COLLECTION

Initially, an extensive bibliographic review was conducted in the La Plata basin complex with the aim of inventorying occurrence records and fish species together with aspects related to the sustainability of natural resources and conservation of ichthyofauna (Araujo & Fontoura, 2024). Technical‐scientific documents and databases of the Global Biodiversity Information Facility (GBIF, 2022) and SpeciesLink (2022) were investigated to access collections containing georeferenced records of the species, allowing confirmation of their occurrence and their characteristics in the Uruguay River basin through the cross‐referencing of information. However, only five occurrence records were obtained for *P. brachyura* in databases and one misidentified record in the Ibicuí River sub‐basin for *P. brachyura* as *P. motoro* was observed in the Fish Collection of the Museum of Science and Technology of the Pontifical Catholic University of Rio Grande do Sul (MCP). Thus, it was decided to collect and use occurrence records entirely obtained from direct investigations with fishermen, with full coverage of the aquatic ecoregions of the Uruguay River basin as proposed by Barradas et al. (2012).

The expeditions approached fishermen from the states of Rio Grande do Sul and Santa Catarina in Brazil, the provinces of Entre Ríos, Corrientes and Misiones in Argentina and fishermen from the departments of Artigas, Salto, Paysandú, Río Negro, Soriano, Flores, Durazno, Tacuarembó and Rivera in Uruguay (2021–2023). It was focused to cover urban areas, highways of all sizes and parties involved with fishery resources, focusing on intersections and roads parallel to water bodies, including rivers, rapids, streams, lakes, hydroelectric plants, dams for agriculture and other reservoirs. Sport fishermen (recreational) and professional fishermen were found in all the locations indicated (Supporting Information S1). The investigations involved oral approaches to the fishermen, including the presentation of cards containing images of confirmed specimens of *P. brachyura* and *P. motoro* together with other freshwater stingrays and marine rays, aiming to eliminate those who did not know how to identify the species that occur in the study area.

The images were arranged in printed and digital format, aiming to facilitate the visualization by the fishermen involved, where questions were promptly clarified and annotated in real time.

**FIGURE 3:**
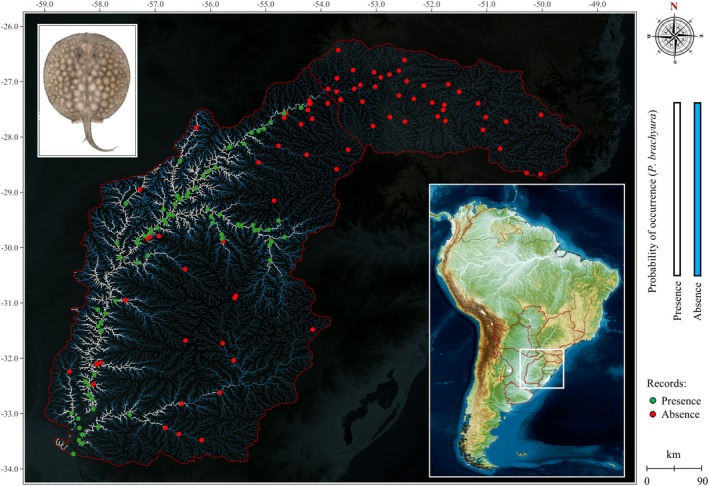
Statistical model for the distribution of the giant short‐tailed river stingray *Potamotrygon brachyura* in the upper Uruguay and lower Uruguay, as predicted by logistic regression models (binary gradient in white for *p* < 0.50), and observed in the field through investigations with fishermen (green dots = presence, and red dots= absence).

## 4 GEOPROCESSING AND CONSTRUCTION OF PROBABILISTIC MODELS

Presence and absence records of stingrays in the ecoregions had their geographical coordinates precisely corrected to the channel of water bodies using raster images of topography and bathymetry from global models (GEBCO, 2023). Other environmental descriptors, apart from elevation [*a*_1_, the height (altitude) above mean sea level], such as upstream distance [*a*_2_, the distance between the most borderline upstream point in the river network (watershed division) and the occurrence record location), downstream distance [*a*_3_, the distance between the most borderline downstream point in the river network (Río de La Plata estuary) and the occurrence record location], occurrence above or below the Salto de Yucumã (*a*_4_, occurrence record location in lower or upper Uruguay FEOW, respectively) and drainage areas (*a*_5_, runoff function), were obtained from adaptations of Lehner and Grill (2013) updated via the HydroSHEDs (2024) database and subjected to rasterization. All data were log transformed (natural logarithms), except those above and below the Salto do Yucumã. The capture of transformed data was based on maps and records of presence (1) or absence (0) of each species. Absence records between sections of presence were excluded for *P. motoro* analysis but remained highlighted in the mappings. For the elaboration of mathematical models, the information obtained was subjected to logistic regression analyses using the Backward (Wald) elimination function for removal of independent variables (*p* > 0.05) using the SPSS application software version 17.5. These models were plotted in the QGIS Raster calculator.

**FIGURE 4:**
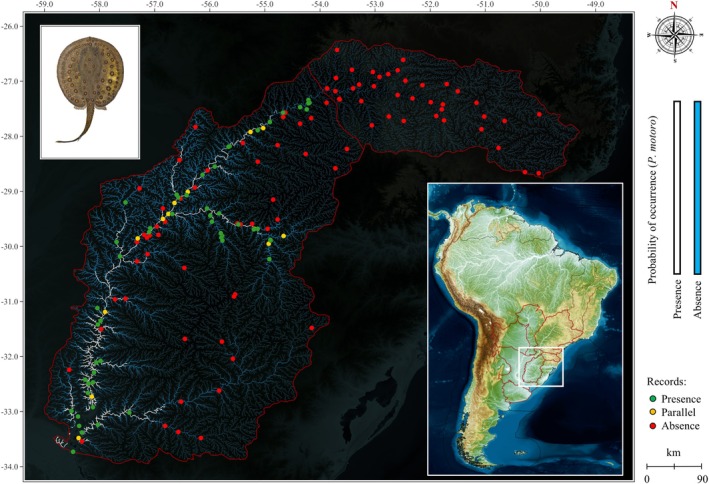
Statistical model for the distribution of the motoro freshwater stingray *Potamotrygon motoro* in the upper Uruguay and lower Uruguay, as predicted by logistic regression models (binary gradient in white for *p* > 0.50) and observed in the field through investigations with fishermen (green dots = presence, yellow dots = excluded parallel records declared as absence within presence records, and red dots = absence).

## 5 RESULTS

In total, 129 validated surveys were conducted; the majority composed of males (97.7%). Ages ranged between 14 and 86 years, with the greatest participation from ages between 60 and70 years (42.6%) followed by those between 21 and 40 years (37.2%). The time of experience had the greatest representativeness between 21 and 40 years of fishing (37.5%), where the experiences of 1 to 20 years and 40 to 60 years of practices with catches had the same proportions (27.3%). In sites with more than one simultaneous interview or notes from many water bodies in small areas, a consensus was chosen between answers and a synthesis of notes. The capture locations (156) were obtained from GPS systems and were later plotted using QGIS as occurrence records between presence and absence for *P. brachyura* and *P. motoro* individually.

According to the interviewees' accounts, there was a consensus on the historical absence of the species in the upper Uruguay River and on the more frequent and abundant presence of freshwater stingrays in the past compared to the present in the lower Uruguay River. The giant or short‐tailed *P. brachyura* is the most ‘common’ species in the Uruguay River and its tributaries, including reports of smaller specimens in streams, wetlands and small lakes. The motoro stingray *P. motoro* appeared to have a less abundant distribution in some sections where there were many reports of *P. brachyura*. Specimens of *P. brachyura* and *P. motoro* were immediately identified by the interviewees with and without the image references presented. However, both species were promptly rejected in at least 11 cases in different locations, where the interviewees' accounts converged on the description of a possible third species in the Uruguay River basin, with dark to black pigmentation and absence of striae, reticules or ocelli, here treated as *Potamotrygon* sp. 1. Many of the interviewees had photos and videos of these animals on their smartphones, except for *Potamotrygon* sp. 1, which was not identified. Reports of typical megafauna specimens weighing more than 30 kg or measuring more than 1 m in disc width (He et al., 2017), as well as slaughters and accidents involving stingrays, were not uncommon.

### 5.1 Giant short‐tailed river stingray *P. brachyura*

Interviewees report that the species has remained present over the years (90%), with some alarming about a decrease in abundance and catch sizes compared to the past. Catches are recent (2016–2023), about 80% occurred between 2021 and 2023. They are considered frequent by 60% of fishermen, in addition to 20% as occasional and, on a smaller scale, 15% as rare. The reports indicate animals weighing an average of 15 kg and measuring around 50 cm in disc width, with the largest records observed with a 120‐kg specimen and another reported with a disc width of over 150 cm. Among the interviewees, 85% report catches by hook and 45% by gillnet, with two reports of accidental fishing with cast‐nets. The baits used for the capture of these animals, called ‘carnadas’ in Spanish, are usually composed of smaller fish, mainly lambari, and a variety meat or organ of butchered animals, except for snails and other organisms as bait at some times. In the case of gillnets, the reported meshes were between 6 and 9 cm between opposite knots. Fishermen report fishing at night/dawn (25%), morning (35%), afternoon (35%) or commonly throughout the day (15%), with mentions of greater occurrences in twilight periods. About half of the interviewees agreed that catches generally occur in summer, whereas another 15% said they occur throughout the year, 10% in spring and only one reported fishing in winter. At least 40% of fishermen reported slaughtering the stingrays and 50% said they released the animals – in both cases, there are reports of tail mutilation due to the presence of stingers.

Specimens of *P*. *brachyura* were only recorded in the Uruguay River and its tributaries downstream of the Salto de Yucumã and, more specifically, below the mouth of the Buricá River (Rio Grande do Sul state, Brazil) (Figure 3). From this region, occurrences extended throughout the Uruguay River and into the mouths of its tributaries to the mouth of the Rio de la Plata, including reports outside the lower Uruguay (Buenos Aires Province, Argentina and Colonia Department, Uruguay). The Ibicuí River, a tributary of the middle Uruguay River, was particularly noteworthy, where records extended into tributaries bordering the upper Uruguay ecoregion and the central portions of the Rio Grande do Sul state. Absences were confirmed in the upstream stretches of all sub‐basins and in front of hydroelectric dams, except for the Salto Hydroelectric Power Plant on the Uruguay River between the cities of Concordia (Entre Ríos, Argentina) and Salto (Salto, Uruguay).

Given the presence and absence observed in the field, the mathematical models showed high effectiveness in predicting the species in the ecoregions, with only 0.6% reduction after the removal of the independent variables of basin area and Salto do Yucumã in three steps (Table 1). The sensitivity rate (true presences) had a drop of only 1.2%, and the specificity (true absences) remained unchanged. The values obtained in R^2^ Nagelkerke indicate good explanatory power of the logistic regression model to the implications in the dependent variable. The estimates of the independent variable (beta coefficient, ‘Estimate’), the standard error values (SE) and Wald along with the significance demonstrate the strength, accuracy and significance of the elevation and distance upstream or downstream variables on the dependent variable (Table 2).

**TABLE 1:** Accuracy of geographical distribution models for giant short‐tailed river stingray *Potamotrygon brachyura* in the Uruguay River basin, considering only elevation data (*a*_1_) and distances upstream (*a*_2_) and downstream (*a*_3_) of the drainage.^a^

	Total	Sensibility	Specificity	False positive	False negative	R^2^ Nagelkerke
Step 1	92.3	97.5	86.8	1.3	6.4	0.693
Step 2	91.7	96.3	86.8	1.9	6.4	0.667
Step 3	91.7	96.3	86.8	1.9	6.4	0.663

^a^ Total of 156 records for *P. brachyura*: 80 for presence and 76 for absence.

**TABLE 2:** Estimated parameters of the logistic equation for the probability of presence (P) of Potamotrygon Brachyura in the Uruguay River Basin.

	Variable	Estimate	SE	Wald	Significance
Step 1	^*a*^_0_	3.057	2.999	1.039	0.308
	^*a*^_1_	−6.824	1.807	14.269	0.000
	^*a*^_2_	1.751	0.768	5.191	0.023
	^*a*^_3_	4.112	1.246	10.896	0.001
	^*a*^_4_	−19.137	5707.219	0.000	0.997
	^*a*^_5_	−5.908	5.299	1.243	0.265
Step 2	^*a*^_0_	3.350	3.299	1.031	0.310
	^*a*^_1_	−8.820	1.636	29.063	0.000
	^*a*^_2_	1.416	0.752	3.543	0.060
	^*a*^_3_	5.157	1.187	18.887	0.000
	^*a*^_4_	−4.525	5.635	0.645	0.422
Step 3	^*a*^_0_	1.498	1.293	1.344	0.246
	^*a*^_1_	−8.830	1.660	28.308	0.000
	^*a*^_2_	0.854	0.374	5.219	0.022
	^*a*^_3_	4.932	1.160	18.070	0.000

*Notes*: *P* = e^(*a*^_0_^+ *a*^_1_^.ln(El)+ *a*^_2_^.ln(DU)+ *a*^_3_^.ln(DD)+ *a*^_4_^.ln(SY)+ *a*^_5_^.ln(BA))^/1 + e ^(*a*^_0_^+ *a*^_1_^.ln(El)+ *a*^_2_^.ln(DM)+ *a*^_3_^.ln(DJ)+ *a*^_4_^.ln(SY)+ *a*^_5_^.ln(BA))^, where El is the elevation given in metres, DU is the distance upstream and DD is the distance downstream, both in kilometres, SY is the Salto do Yucumã with binary occurrence located above or below its locality, BA is the basin area in km^2^, and a_0_ represents the occurrence constant that is not explained by any environmental descriptor, whereas the increment rates in the probability of occurrence are represented by *a*_1_ (elevation), *a*_2_ (distance), *a*_3_ (distance downstream), *a*_4_ (occurrence located above or below the Salto do Yucumã) and a_5_ (basin area). All data, except those above and below the Salto do Yucumã, were log transformed (natural logarithms). A total of 156 records were obtained for *P. brachyura*: 80 for presence and 76 for absence.

Abbreviation: SE, standard error.

**TABLE 3:** Accuracy of geographical distribution models for motor stingray *Potamotrygon motoro* in Uruguay River basin, considering elevation data (*a*_1_), distances upstream (*a*_2_) and downstream (*a*_3_) of the drainage.^a^

	Total	Sensibility	Specificity	False positive	False negative	R^2^ Nagelkerke
Step 1	81.0	71.7	86.5	10.6	8.5	0.572
Step 2	77.5	62.3	86.5	14.1	8.5	0.539
Step 3	76.8	62.3	85.4	14.1	9.2	0.535

^a^ Total of 142 *P. motoro* records: 53 for presence and 89 for absence (14 excluded).

**TABLE 4:** Estimated parameters of the logistic equation for the probability of presence (P) of Potamotrygon motoro in the Uruguay River Basin.

	Variable	Estimate	SE	Wald	Significance
Step 1	^*a*^_0_	1.557	1.785	0.761	0.383
	^*a*^_1_	−4.163	1.466	8.062	0.005
	^*a*^_2_	1.436	0.665	4.670	0.031
	^*a*^_3_	2.262	1.086	4.341	0.037
	^*a*^_4_	−19.069	6008.345	0.000	0.997
	^*a*^_5_	−4.128	3.761	1.204	0.272
Step 2	^*a*^_0_	1.570	1.789	0.770	0.380
	^*a*^_1_	−5.842	1.349	18.756	0.000
	^*a*^_2_	1.166	0.635	3.377	0.066
	^*a*^_3_	3.190	1.040	9.406	0.002
	^*a*^_4_	−2.936	3.722	0.622	0.430
Step 3	^*a*^_0_	0.656	1.104	0.353	0.553
	^*a*^_1_	−5.798	1.363	18.105	0.000
	^*a*^_2_	0.746	0.350	4.545	0.033
	^*a*^_3_	2.951	1.009	8.558	0.003

*Notes: P* = e ^(*a*^_0_^+*a*^_1_^.ln(El)+ *a*^_2_^.ln(DU)+*a*^_3_^.ln(DD)+*a*^_4_^.ln(SY)+*a*^_5_^.ln(BA))^/1 + e ^(*a*^_0_^+*a*^_1_^.ln(El)+*a*^_2_^.ln(DM)+ *a*^_3_^.ln(DJ)+*a*^_4_^.ln(SY)+*a*^_5_^.ln(BA))^, where El is the elevation given in metres, DU is the distance upstream and DD is the distance downstream, both in kilometres, SY is the Salto do Yucumã, with binary occurrence located above or below its locality, BA is the basin area in km^2^, and *a*_0_ represents the occurrence constant that is not explained by any environmental descriptor, whereas the increment rates in the probability of occurrence are represented by *a*_1_ (elevation), *a* _2_ (distance), *a*_3_ (distance downstream), *a*_4_ (occurrence located above or below the Salto do Yucumã) and *a*_5_ (basin area). All data, except those above and below the Salto do Yucumã, were log transformed (natural logarithms). A total of 142 *P. motoro* records were obtained: 53 for presence and 89 for absence (14 excluded).

Abbreviation: SE, standard error.

### 5.2 Motoro freshwater stingray *P. motoro*

Among fishermen, there was agreement that the species is currently present (92%). The latest reports of catches have all occurred in the past five years. The species is considered rare in 46% of the approaches, as well as occasional or frequent in 23%, respectively, and may vary based on the region or time of the year. The observed sizes are substantially different from those obtained for *P. brachyura*; the average for *P. motoro* was 4 kg, with the highest recorded weight at 20 kg and the largest size at approximately 100 cm in disc width (considered atypical by the interviewees). The use of hooks and gillnets as fishing gear was simultaneously reported by 77% and 46% of the interviewees, respectively. The baits included fish, molluscs and a variety of meat and organs (offal). The gillnets were reported to have mesh‐size of 7 to 9 cm between opposite knots. Fishing was reported at night and dawn (38%), morning (46%), afternoon (15%) or regardless of time (15%). The most common period for fishing the species was in summer (46%). About half of the interviewees (46%) reported slaughtering under some circumstances.

The presence of *P. motoro* was announced in stretches immediately below the Salto de Yucumã, unlike what was observed for *P. brachyura*, whose distribution intensifies from the regions around the mouth of the Santa Rosa River (Figure 4). According to the personal observations of the fishermen investigated, except for the Ibicuí River, stretches where *P. brachyura* is prominent, *P. motoro* appears to be rare or even non‐existent, as was observed in the stretches between the mouth of the Ibicuí and Quaraí rivers in the Paso de Los Libres region (Corrientes Province, Argentina) and Uruguaiana (State of Rio Grande do Sul, Brazil). Apparently, these animals are more abundant and frequent in smaller courses.

**FIGURE 5:**
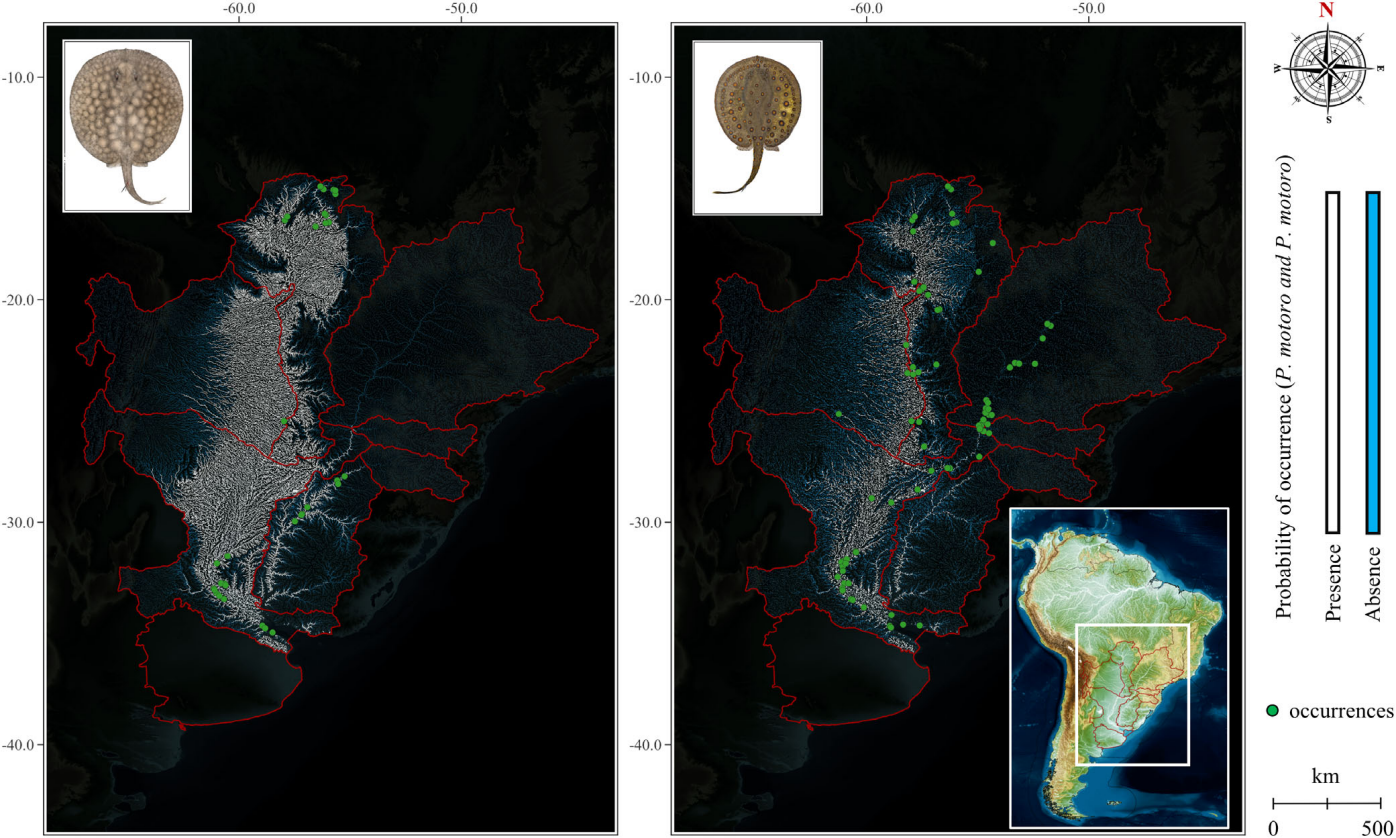
Statistical model for the distribution of the freshwater stingrays *Potamotrygon brachyura* (left) and *Potamotrygon motoro* (right) for upper Uruguay and lower Uruguay as predicted by logistic regression models (binary gradient in white for *p* > 0.50) extrapolated to La Plata basin. Green dots represent occurrences recorded in La Plata basin from the Global Biodiversity Information Facility (GBIF) and SpeciesLink (2024) for *P. brachyura* (74) and *P. motoro* (252). Lower Uruguay only counts 15 occurrences for *P. brachyura* and without occurrences for *P. motoro*. The upper Paraná occurrences of *P. motoro* is attributed to non‐native exemplars of *Potamotrygon amandae* originated from the fish passage ‘Canal de Piracema’ at the Itaipu Dam (Ota et al., 2018). Above Itaipú only *Potamotrygon falkneri* and *P. amandae* occur, with *P. amandae* standing out. Note that the estimated presence is ranging from a low distribution for Uruguay ecoregion to a high distribution for entire La Plata due the use of elevation, distance upstream and downstream variables. As observed in Lucifora et al. (2015), the use of other variables can improve these estimates (Tables 2 and 4).

The statistical models obtained slightly lower results than those observed for *P. brachyura* (Table 3). The mathematical differences in the global (total) rate occurred in 3.5% from the first to the second step and 4.2% from the first to the third step (Table 4). However, even with the decrease in sensitivity, the specificity remained constant, with R^2^ Nagelkerke values explaining the influence of the independent variables on the dependent variable. As can also be observed for *P. brachyura*, even with relatively high B values, larger SEs and smaller Wald values involving the basin area and the Salto de Yucumã implied lower statistical significance and, consequently, the exclusion of these variables in the probabilistic model of presence and absence for *P. motoro*. The information obtained from the knowledge of fishermen in the Uruguay River basin resulted in a reduced estimative of *P. motoro* relative to *P. brachyura* not only in the ecoregions of the Uruguay River but also in the entire La Plata basin complex (Figures 4 and 5).

### 5.3 Unidentified freshwater stingray *Potamotrygon* sp. 1

Based on identifications made by different fishermen, which resulted in 11 presence records, the latest catch reports occurred in the past 5 years, as also observed for *P. motoro*. The taxon appeared to be rarer or occasional in seven approaches. The average size was 2.5 kg. This is a much smaller average than that observed for *P. brachyura* (15 kg) and *P. motoro* (4 kg), where the largest specimen supposedly belonging to this taxon would be around 10 kg, according to a single interviewee. About eight catches were made by hook. All these catches were made with bait using fish parts or small fish. There did not appear to be a specific time of day for the catch. The catches occurred all year round, with greater chances expected in the summer due to a common sense among fishermen regarding the greater piscivory in this season. Only one interviewee reported slaughtering these specimens; more commonly, they were released due to their small size. All attempts to identify the species through further investigations with fishermen were inconclusive. Without a captured specimen, the observed characteristics can easily be confused with those of other specimens occurring in the Paraná‐Paraguay region, particularly as potamotrygonids may exhibit different phenotypes and adaptations depending on the habitat, life stage and other factors.

## 6 DISCUSSION

According to phylogenetic investigations on the biogeography of Neotropical stingrays (Potamotrygonidae), paleogeographic changes have profound influences on the biodiversity evolution of these and other organisms on a continental scale (Fontenelle et al., 2021). Also known as ‘peripheral division’ taxa (Reis et al., 2016), these taxa are involved in marine invasions that occurred during the Oligocene and Miocene and would have given rise to freshwater stingrays from the upper Amazon region in the Pebas wetland systems (Fontenelle et al., 2021). Thus, the colonization of adjacent areas was driven by factors that influenced the ecology of these animals. Evidence of Pliocene colonization from incursions from the upper Amazon into the Paraná‐Paraguay basins, where the Uruguay River should be included, would explain the greater species richness due to the greater persistence of older lineages and higher speciation rates, along with the export of taxa to more recent and, consequently, more biodiverse areas. In this context, the connections between the headwaters of the Amazon (such as the Madeira) and La Plata (such as the Bolivian Chaco) basins would have played an important role during the Miocene, including through fossil and geological evidence (Albert et al., 2018). A curious aspect is that *P. brachyura*, in addition to being the largest freshwater stingray in the Neotropical region, represents the oldest lineage recovered from the Paraná‐Paraguay systems, dating back approximately 12 million years and colonized from the Amazon (Fontenelle et al., 2021). In this context, two of these genomic samples include the Uruguay River basin: the most distant region of original dispersion and the least investigated as well.

Freshwater stingrays have never been studied exclusively wihin the Uruguay River ecoregions. With the exception of Oddone et al. (2012) (who formally described the occurrences of *P. brachyura* and *P. motoro* in the Uruguay River and its tributaries) and Lucifora et al. (2015) (who advanced understanding of the geographical distribution and demonstrated the importance of conservation of *P. brachyura* in the Paraná‐Paraguay and Uruguay river basins), freshwater stingrays were only cited and briefly described among other taxa in publications that listed inventories of freshwater fauna. Historically, multiple publications included the occurrence of *P. histrix* in the Uruguay River (Carrera, 1976; Devincenzi & Teague, 1942; Nión et al., 2016), including in periods that different parts consider only the giant short‐tailed stingray *P. brachyura* and the stingray *P. motoro* for both western and eastern sides (Litz & Koerber, 2014; Loureiro Barrella et al., 2023; Sverlij et al., 1998). In the review of the *P. motoro* species complex carried out by Loboda et al. (2013) in the Paraná‐Paraguay axis systems, *Potamotrygon pauckei* Castex, 1963 and *Potamotrygon labradori* Castex, Maciel & Achenbach, 1963 were synonymized with *P. motoro*, whereas *Potamotrygon alba* Castex, 1963 was considered a nomen dubium in agreement with previous authors. The investigations resulted in two new ocellated species very similar to *P. motoro*: *Potamotrygon pantanensis* Loboda & Carvalho, 2013 and *Potamotrygon amandae* Loboda & Carvalho, 2013. After the description, *P. amandae* also became part of the lower Paraná ecoregion in Argentina, together with *P. brachyura*, *P. motoro* and *P. histrix*, in addition to at least one unidentified species (Almirón et al., 2015). Although the Uruguay River ecoregions are densely sampled on the Brazilian side (Bertaco et al., 2016), and there is consensus on the presence of *P. motoro* in Argentina and Uruguay, the lack of investigations into these taxa and their congeners in these areas demands the need for new systematic and biogeographical studies. Pereira et al. (*in prep*.) point out that, although latitudinal, topographic and spatial gradients tend to favour greater species richness and taxonomic composition, the lower Paraná ecoregion records 270 species compared to 295 species in the lower Uruguay (GBIF, 2022; SpeciesLink, 2022). The sum of factors in these scenarios reinforces the need for more sampling and investigations on freshwater stingrays not only in the lower Uruguay but also in substantial portions of La Plata basin (Figure 5).

The logistic model of geographical distribution for *P. brachyura* proved to be highly efficient not only in the tabulated statistical parameters but also in the graphic expression in predicting presence and absence, yielding results similar to those obtained by Lucifora et al. (2015) in the main channel and other water bodies in low lands. It is possible to verify that the predicted data coincide with the observed data, including higher probabilities of occurrence from the mouth of the Uruguay River to the Río de la Plata estuary downstream to the Salto de Yucumã. Additionally, the highest probabilities of occurrence extend to the less‐elevated portions of the main tributaries of the lower Uruguay. In this context, some values differ from those previously found by Lucifora et al. (2015), and an important point is the Ibicuí River. Previously, although they excluded the only occurrence of *P. brachyura* in the Ibicuí River considering it putative, rates were obtained that indicated the presence of the species in this water body. Here, the Ibicuí River and the lower reaches downstream of its tributaries obtained prediction rates close to 100%, where field reports confirmed a very abundant and frequent presence in the Ibicuí River and, in some cases, declared to be greater than in the Uruguay River itself. One association made by the fishermen was the nature of its sandy substrate.

Previously, it was predicted that very high presence rates would also be upstream of the Rincón del Bonete reservoir (Department of Tacuarembó, Uruguay) (Lucifora et al., 2015). Here, there was no observed confirmation of the species beyond the ‘Central Hidroeléctrica Constitución’ reservoir or ‘Palmar Dam’, where rates drop abruptly downstream of Rincón del Bonete. These facts are most likely due to the more widely distributed data coverage of the Paraná‐Paraguay‐Uruguay basin carried out by Lucifora et al. (2015) compared to a more concentrated and detailed coverage obtained in this study for the Uruguay River ecoregion, and there is still a small increase in the number of presence records (10). Another factor would be the use of a larger number of independent variables, positively associated with the accumulation of flow, percentage of open water and submerged vegetation and floodplain ecoregions, negatively associated with the humidity index and altitude, culminating in six different variables (Lucifora et al., 2015). On the contrary, in the present study, only three independent variables were used, positively associated with upstream distance values and negatively with downstream distance and elevation. However, although very precise for the Uruguay River ecoregions, the prediction model obtained here provides a virtual distribution of *P*. *brachyura* for the low‐altitude water bodies present in the lower Paraná, Chaco and Paraguay, especially in the wetlands of the Paraná River Delta to the Pantanal. Thus, its applicability to neighbouring ecoregions of the same water complex should be evaluated, as it represents an excellent advantage in reducing sampling, and can be adapted through the insertion of more independent variables, such as the success obtained by Lucifora et al. (2015).

*P. motoro* demonstrated a well‐defined graphical prediction for both the upper and lower Uruguay ecoregions and the lower Paraná, Chaco, Paraguay and, curiously, the upper Paraná, although presenting slightly lower values in statistical metrics compared to *P. brachyura*. In the upper Paraná, the probability of occurrence in the main river and its tributaries was between 20% and 30%. An important fact, given that the Salto de Yucumã represents an important biogeographical barrier for the retention of *P. brachyura* and *P. motoro* in the lower Uruguay, as observed here, the now‐extinct Sete Quedas prevented the dispersal of rays and other fish from the lower Paraná to the upper Paraná (Loboda et al., 2013; Ota et al., 2018; Dos Reis et al., 2020). With the construction of the Itaipu Hydroelectric Power Plant next to the ‘Canal de Piracema’ (a channel for fish transposition), the species previously considered part of the *P. motoro* complex together with the *Potamotrygon* cf. *falkneri* Castex, Maciel, 1963 and *Potamotrygon schuhmacheri* Castex 1964 (Garrone Neto & Haddad Junior, 2010) would have started to integrate into the upper Paraná systems. However, after the review by Loboda et al. (2013), the occurrence of *P. amandae* and the possible occurrence of *P*. cf. *falkineri* (Ota 2018) in the upper Paraná were confirmed. This could discreetly explain the ambivalence observed here for *P. motoro*: there are low‐to‐medium probability rates of occurrence in the portions of the upper Uruguay and lower Paraná, but there are no valid occurrence records for the species today. However, the taxonomic proximity shared by this group must be respected in the context of the spread of non‐native species, as *P. motoro* has already been declared an established species in Southeast Asia in Singapore (Ng et al., 2010) and Indonesia (Jerikho et al., 2023) within other stingrays, reinforcing the need for further evaluations of this taxon within and outside the lower Uruguay. The predicted geographical distribution for *P. motoro* here coincides very well with the data obtained by Lucifora et al. (2015) for *P. brachyura*, reinforcing the effectiveness of the model and the ecological and evolutionary proximities of the diversity of stingrays in the La Plata basin, as observed by Fontenelle et al. (2021).

The possible occurrence of a third taxon in the lower Uruguay River is intriguing. Reports with no connection from the north, west and centre of the Rio Grande do Sul state in Brazil and one in the northwest of Uruguay point to the same characteristics: dark (even black) and without any apparent spots, besides being rarer and sometimes smaller than the other confirmed stingrays, according to fishermen. These phenotypes and occurrence trends, theoretically, would not fully coincide with any of the two observed species. It is known that specimens of *P. brachyura* can present dark colouration and that specimens of the *P. motoro* complex can also present darker phenotypes and less‐visible ocelli, as observed in *P. amandae* (Loboda & Carvalho, 2013). It is also known that freshwater stingrays tend to change colour in camouflage, stress, feeding, reproduction processes, etc. (personal observation), which would explain the confirmation of only two species. However, the existence of multiple untraceable documents citing the supposed presence of a third species in the Uruguay River, including reports by the State Foundation for Environmental Protection of Rio Grande do Sul (FEPAM) (Querol et al., 2018), added to the absence of specimens deposited in collections, prevents a fully theoretical conclusion. Another factor to be considered is the historical connection of the lower Uruguay with the lower Paraná in addition to the Río de la Plata, with important communications between one basin and the other through the Miriñay River, whose source is in the wetlands shared with the lower Paraná in the Esteros del Ibirá, where other species of stingrays occur (Almirón et al., 2015) and high rates of prediction of presence of *P. brachyura* and *P. motoro*. Thus, the attempt to capture animals in potential occurrence points to determine a third taxon distinct from *P. brachyura* and *P. motoro* must be considered.

Considering logistic models for predicting large species of greater importance among the fishery resources in the upper and lower Uruguay River, *P. brachyura* showed a much smaller geographical distribution than that obtained by Barradas et al. (2012) for the spotted surubim *P. corruscans*, dourado *S. brasiliensis*, grumatã *Prochilodus lineatus* (Valenciennes 1837) and piava *Megaleporinus obtusidens* (Valenciennes 1837). As observed for *P. brachyura*, *P. motoro* also presented a reduced geographical distribution compared to the large migratory species of the Uruguay River (Barradas et al., 2012). This can be potentially worrying, considering the sum of environmental impacts scaled by agriculture and animal production, industry, urbanization, ecotoxicological contamination and the accumulation of established and planned dams, in addition to fishing itself on a smaller scale (Lucifora et al., 2015; CIC/OAS, 2017). Large migratory fish in the lotic ecosystems, and even in semilentic/lentic environments with dams, are representatives among the fishery resources, along with some other lentic species (Agostinho et al., 2007; Barradas et al., 2012; Ministry of Aquaculture and Fisheries, 2011). However, it is already possible to notice imminent fishing pressures on freshwater stingrays in the Uruguay River drainages, both by commercial/subsistence fishing and by sport fishing, which may also include the slaughter of animals (Lucifora et al., 2015).

Driven by the aquarium and ornamental fish market, different species have been proposed and included for trade restrictions by South American countries in CITES (2024). However, in addition to there being no proven evidence that this activity has a greater influence on threats to populations compared to other anthropogenic changes, such as hydroelectric and agricultural dams, natural cover loss and pollution, these political‐environmental strategies, together with the tightening of legislation, represent little practical resolution due to the deficient monitoring. In addition, it can increase further marginalizing fishing populations already in a situation of socioeconomic vulnerability (Agostinho et al., 2007). During the expeditions, it was not uncommon to observe greater damage caused by sport fishing along the Uruguay River drainages due to the disproportionately greater financial power of its practitioners compared to commercial/artisanal fishing. This includes access to larger fishing areas through private properties on the banks of the rivers, the use of large land vehicles, boats, highly efficient fishing gear, freezers for fish storage and firearms for hunting. It has an apparent intensification on the eve of the spawning season and during legal fishing bans on the reproductive period, including the occurrence of parallel activities. On the contrary, reports of overfishing by commercial fishing were not uncommon. The permissiveness in the acquisition of fishing licences was also a common point of discussion among fishermen of different modalities, along with the indications of little or highly targeted inspections. However, complaints involving the abusive use of pesticides and the diversion of water for irrigation of crops were discussed by both professional and amateur fishermen, especially in the State of Rio Grande do Sul, Brazil and in different Departments of Uruguay.

## 7 CONCLUSION

Investigations conducted through fishers' knowledge and the ecosystem approach to fisheries have proven sufficient for obtaining data on biodiversity, as well as potentially on the socioeconomic and political‐environmental panoramas. Logistic models obtained here from a sub‐basin or ecoregion demonstrate that the use of smaller areas can be adapted for extrapolations in larger drainages and entire basins complexes, thus reducing financial costs and other technical‐scientific impediments. However, new investigations should be conducted in the lower Uruguay to sample for possible new species, and new approaches are also recommended for the ecoregions of the lower Paraná, Chaco, Paraguay and upper Paraná.

## AUTHOR CONTRIBUTIONS

Danilo Araujo Soares Pereira contributed to the conception and design of the project, manuscript preparation, field expeditions, scientific illustrations, mapping, data generation, and data analyses. Roberto E. Reis contributed through ideas, supervision, data analysis, and critical review. Nelson F. Fontoura contributed to the conception and design of the project, ideas, mapping, statistical analyses, mathematical models, supervision, and critical review.

## FUNDING INFORMATION

This study was supported by the Coordination for the Improvement of Higher Education Personnel (CAPES), Ministry of Education (MEC, Federal Government of Brazil), under Finance Code 001.

## CONFLICT OF INTEREST STATEMENT

In accordance with Wiley's policy and our ethical responsibilities as researchers, we declare that we have no financial, commercial or entrepreneurial conflicts of interest that could influence the research presented in the enclosed manuscript. Additionally, we have an approved plan in place to manage any potential conflicts that may arise in connection with this study.

## Supporting information


**Data S1.** Supporting information.
