# Supplementary material for: Distribution modelling for Neotropical freshwater stingrays Potamotrygon brachyura and Potamotrygon motoro (Myliobatiformes, Potamotrygonidae) in the Uruguay River basin
Source: J Fish Biol. 2025 Sep 11;108(1):43–56. doi: 10.1111/jfb.70219 (PMC13033953; doi:10.1111/jfb.70219)
Supplement: Supplementary file 1 — Data S1. Supporting information. [file JFB-108-43-s001.docx]

**Supplementary material.** Records of presence (1) and absence (0) of the freshwater stingrays *Potamotrygon brachyura*, *P. motoro* and *P.* sp. 1 in the FEOW of the Uruguay River basin. Elevation (a1), upstream (a2), downstream (a3), Yucumã (a4), and basin area (a5) data obtained from HydroSHEDS (2023).

| N | Water bodies and localities | Latitude (W) | Longitude (S) | Elevation (m) | Upstream (km) | Downstream (km) | Yucumã (0) | Basin (km²) | *P. brachyura* | *P.  motoro* | *P.* sp. |
| --- | --- | --- | --- | --- | --- | --- | --- | --- | --- | --- | --- |
|  |  |  |  |  |  |  |  |  |  |  |  |
| 1 | Río Uruguay (ER, ARG/ SO, URY) | -33,734 | -58,475 | 0 | 0,00 | 0,00 | 0 | 0,00 | 1 | 1 | 0 |
| 2 | Río Negro (SO, URY) | -33,381 | -58,323 | 0 | 775,70 | 0,90 | 0 | 6,99 | 1 | 1 | 0 |
| 3 | Río San Salvador (SO, URY) | -33,536 | -58,320 | 3 | 129,80 | 8,40 | 0 | 5,59 | 1 | 0 | 0 |
| 4 | Río Uruguay (ER, ARG/ SO, URY) | -33,480 | -58,385 | 0 | 137,90 | 0,00 | 0 | 5,63 | 1 | 0* | 0 |
| 5 | Río Negro (SO/ RN, URY) | -33,023 | -57,460 | 17 | 634,20 | 142,40 | 0 | 6,94 | 1 | 1 | 0 |
| 6 | Rio Negro (SO/ RN, URY) | -33,241 | -58,031 | 3 | 731,40 | 45,20 | 0 | 6,99 | 1 | 1 | 0 |
| 7 | Río Uruguay (ER, ARG/ RN, URY) | -33,258 | -58,375 | 0 | 0,00 | 0,00 | 0 | 0,00 | 1 | 1 | 0 |
| 8 | Río Queguay (PY, URY) | -32,076 | -57,985 | 14 | 256,50 | 256,50 | 0 | 6,04 | 0 | 1 | 0 |
| 9 | Río Queguay (PY, URY) | -32,109 | -58,060 | 2 | 261,60 | 109,50 | 0 | 6,06 | 0 | 1 | 0 |
| 10 | Arroyo Negro (RN, URY) | -32,475 | -58,125 | 5 | 98,80 | 60,00 | 0 | 5,44 | 0 | 1 | 0 |
| 11 | Río Uruguay (ER, ARG/ RN, URY) | -32,923 | -58,118 | 2 | 2060,10 | 0,00 | 0 | 7,55 | 1 | 1 | 0 |
| 12 | Arroyo Gualeyán (ER, ARG) | -32,991 | -58,505 | 3 | 180,70 | 11,60 | 0 | 5,94 | 1 | 1 | 0 |
| 13 | Río Gualeguaychú (ER, ARG) | -33,089 | -58,392 | 0 | 192,00 | 0,00 | 0 | 5,98 | 1 | 1 | 0 |
| 14 | Río Uruguay (ER, ARG) | -32,485 | -58,196 | 0 | 2021,60 | 38,80 | 0 | 7,54 | 1 | 1 | 0 |
| 15 | Arroyo del Molino (ER, ARG) | -32,418 | -58,261 | 6 | 31,90 | 60,50 | 0 | 3,68 | 1 | 1 | 0 |
| 16 | Arroyo Osuna (ER, ARG) | -32,661 | -58,210 | 2 | 28,70 | 25,70 | 0 | 4,15 | 1 | 1 | 0 |
| 17 | Río Uruguay (ER, ARG/ RN, URY) | -32,731 | -58,144 | 2 | 2037,00 | 23,40 | 0 | 7,55 | 1 | 0* | 0 |
| 18 | Río Uruguay (ER, ARG/ PY, URY) | -32,293 | -58,103 | 0 | 1985,50 | 74,90 | 0 | 7,54 | 1 | 1 | 0 |
| 19 | Represa de Salto (ER, ARG/ SA, URY) | -31,193 | -57,899 | 32 | 1833,30 | 227,00 | 0 | 7,51 | 1 | 0* | 0 |
| 20 | Río Daiman (PY, URY) | -31,498 | -57,966 | 9 | 163,80 | 183,40 | 0 | 5,67 | 1 | 0 | 0 |
| 21 | Río Arapey (SA, URY) | -30,957 | -57,719 | 32 | 229,40 | 277,10 | 0 | 6,20 | 1 | 0 | 0 |
| 22 | Río Uruguay (ER, ARG/ SA, URY) | -31,120 | -58,038 | 32 | 28,00 | 239,20 | 0 | 4,56 | 1 | 1 | 0 |
| 23 | Represa de Salto (ER, ARG/ SA, URY) | -31,416 | -58,029 | 3 | 1862,30 | 198,10 | 0 | 7,51 | 1 | 1 | 0 |
| 24 | Rio Uruguai (ER, ARG/ SA, URY) | -31,348 | -57,984 | 2 | 1855,40 | 205,00 | 0 | 7,51 | 1 | 1 | 0 |
| 25 | Arroio Itapitocai (RS, BRA) | -29,791 | -57,189 | 48 | 46,00 | 436,50 | 0 | 4,67 | 1 | 0 | 0 |
| 26 | Río Arapey (SA,URY) | -30,950 | -57,533 | 41 | 206,20 | 300,20 | 0 | 6,19 | 0 | 0 | 1 |
| 27 | Arroio Guaraputan (RS, BRA) | -29,918 | -57,322 | 40 | 1649,30 | 411,00 | 0 | 7,40 | 1 | 0 | 0 |
| 28 | Arroio Touro Passo (RS, BRA) | -29,640 | -56,964 | 48 | 67,20 | 472,30 | 0 | 5,11 | 1 | 0 | 0 |
| 29 | Rio Quaraí (RS, BRA/ AR, URY) | -30,141 | -57,130 | 59 | 20,20 | 445,00 | 0 | 4,11 | 1 | 0 | 1 |
| 30 | Rio Uruguai (RS, BRA/ CR, ARG) | -29,497 | -56,848 | 48 | 1576,50 | 483,90 | 0 | 7,40 | 1 | 0* | 0 |
| 31 | Barragem Sanchuri (RS, BRA) | -29,554 | -56,809 | 50 | 20,90 | 489,60 | 0 | 4,26 | 1 | 0 | 0 |
| N | Water bodies and localities | Latitude (W) | Longitude (S) | Elevation (m) | Upstream (km) | Downstream (km) | Yucumã (0) | Basin (km²) | *P. brachyura* | *P.  motoro* | *P.* sp. |
|  |  |  |  |  |  |  |  |  |  |  |  |
| 32 | Rio Uruguai (RS, BRA/ CR, ARG) | -29,742 | -57,098 | 42 | 1614,60 | 445,80 | 0 | 7,40 | 1 | 1 | 0 |
| 33 | Rio Uruguai (RS, BRA/ CR, ARG) | -30,175 | -57,626 | 39 | 1695,20 | 365,20 | 0 | 7,41 | 1 | 1 | 0 |
| 34 | Rio Quaraí (RS, BRA/ AR, URY) | -30,272 | -57,316 | 49 | 263,70 | 407,60 | 0 | 6,25 | 1 | 0 | 0 |
| 35 | Rio Uruguai (RS, BRA/ CR, ARG) | -29,854 | -57,307 | 43 | 1642,00 | 418,30 | 0 | 7,40 | 1 | 0* | 0 |
| 36 | Arroio Maracatu (RS, BRA) | -29,601 | -55,443 | 73 | 63,90 | 710,60 | 0 | 4,89 | 1 | 0 | 0 |
| 37 | Rio Ibicuí (RS, BRA) | -29,596 | -55,483 | 68 | 354,60 | 706,30 | 0 | 6,60 | 1 | 1 | 0 |
| 38 | Arroio Caraguataí (RS, BRA) | -29,582 | -55,504 | 72 | 36,00 | 706,40 | 0 | 4,48 | 1 | 0 | 0 |
| 39 | Rio Itu (RS, BRA) | -29,396 | -55,831 | 67 | 141,30 | 632,00 | 0 | 5,57 | 1 | 1 | 1 |
| 40 | Rio ibirapuitã (RS, BRA) | -29,888 | -55,775 | 87 | 158,60 | 702,30 | 0 | 5,65 | 0 | 1 | 1 |
| 41 | Rio Ibicuí (RS, BRA) | -29,309 | -56,052 | 57 | 466,20 | 594,70 | 0 | 6,76 | 1 | 0 | 0 |
| 42 | Rio Ibirapuitã (RS, BRA) | -29,755 | -55,799 | 77 | 15,60 | 683,50 | 0 | 5,91 | 1 | 1 | 0 |
| 43 | Rio Santa Maria (RS, BRA) | -29,879 | -54,897 | 81 | 263,50 | 797,40 | 0 | 6,31 | 1 | 1 | 0 |
| 44 | Rio Ibirapuitã (RS, BRA) | -29,837 | -55,793 | 94 | 167,50 | 693,30 | 0 | 5,77 | 1 | 1 | 0 |
| 45 | Rio Ibirapuitã (RS, BRA) | -29,442 | -55,992 | 63 | 234,60 | 626,30 | 0 | 6,03 | 1 | 1 | 0 |
| 46 | Arroio Inhanduí (RS, BRA) | -29,546 | -55,964 | 69 | 85,00 | 644,40 | 0 | 5,12 | 1 | 1 | 0 |
| 47 | Rio Caverá (RS, BRA) | -29,810 | -55,775 | 78 | 173,10 | 687,80 | 0 | 5,29 | 1 | 1 | 0 |
| 48 | Rio Santa Maria (RS, BRA) | -30,234 | -54,915 | 91 | 208,50 | 852,40 | 0 | 6,22 | 1 | 1 | 1 |
| 49 | Arroio Jacaquá (RS, BRA) | -29,689 | -55,203 | 77 | 36,50 | 744,70 | 0 | 4,65 | 1 | 1 | 0 |
| 50 | Arroio Inhacundá (RS, BRA) | -29,592 | -55,234 | 81 | 46,60 | 737,00 | 0 | 4,65 | 1 | 0 | 0 |
| 51 | Rio Jaguari (RS, BRA) | -29,678 | -54,946 | 83 | 189,10 | 777,30 | 0 | 5,79 | 1 | 0 | 0 |
| 52 | Rio Ibicuí (RS, BRA) | -29,684 | -55,194 | 73 | 321,90 | 739,00 | 0 | 6,57 | 1 | 0* | 0 |
| 53 | Rio Cacequi (RS, BRA) | -29,889 | -54,893 | 84 | 91,40 | 802,10 | 0 | 5,40 | 1 | 1 | 1 |
| 54 | Rio Jaguarizinho (RS, BRA) | -29,514 | -54,773 | 94 | 80,80 | 814,80 | 0 | 5,25 | 1 | 0 | 0 |
| 55 | Rio Santa Maria (RS, BRA) | -29,949 | -54,934 | 84 | 249,40 | 808,30 | 0 | 6,25 | 1 | 0* | 0 |
| 56 | Rio Jaguari (RS, BRA) | -29,680 | -55,134 | 79 | 214,60 | 751,90 | 0 | 5,84 | 1 | 1 | 0 |
| 57 | Rio Ibicuí (RS, BRA) | -29,806 | -54,655 | 88 | 170,10 | 816,50 | 0 | 5,86 | 1 | 0* | 0 |
| 58 | Rio Ibicuí (RS, BRA) | -29,311 | -56,052 | 57 | 466,20 | 594,70 | 0 | 6,76 | 1 | 1 | 1 |
| 59 | Rio Ibicuí (RS, BRA) | -29,408 | -56,682 | 45 | 555,70 | 505,20 | 0 | 6,80 | 1 | 1 | 0 |
| 60 | Arroyo Yatay (CR, ARG) | -29,670 | -57,076 | 45 | 19,50 | 459,10 | 0 | 4,57 | 1 | 1 | 0 |
| 61 | Río Guaviraví (CR, ARG) | -29,314 | -56,853 | 53 | 76,10 | 507,10 | 0 | 5,31 | 1 | 0 | 0 |
| 62 | Río Miriñay (CR, ARG) | -29,917 | -57,694 | 47 | 197,50 | 407,50 | 0 | 6,14 | 1 | 1 | 0 |
| 63 | Río Miriñay (CR, ARG) | -29,204 | -57,534 | 47 | 29,40 | 504,20 | 0 | 5,59 | 1 | 1 | 0 |
| 64 | Río Uruguay (CR, ARG) | -29,207 | -56,644 | 52 | 1533,80 | 526,60 | 0 | 7,26 | 1 | 0* | 0 |
| 65 | Río Aguapey (CR, ARG) | -29,060 | -56,595 | 51 | 547,00 | 547,00 | 0 | 5,97 | 1 | 1 | 0 |
| N | Water bodies and localities | Latitude (W) | Longitude (S) | Elevation (m) | Upstream (km) | Downstream (km) | Yucumã (0) | Basin (km²) | *P. brachyura* | *P.  motoro* | *P.  sp.* |
|  |  |  |  |  |  |  |  |  |  |  |  |
| 66 | Río Aguapey (CR, ARG) | -28,430 | -56,545 | 62 | 180,40 | 633,40 | 0 | 5,76 | 1 | 0 | 0 |
| 67 | Barragem de Itaqui “Cambaí” | -29,130 | -56,534 | 50 | 21,20 | 544,40 | 0 | 4,31 | 1 | 0 | 0 |
| 68 | Rio Uruguai (RS, BRA/ CR, ARG) | -29,115 | -56,574 | 45 | 1524,10 | 536,30 | 0 | 7,26 | 1 | 1 | 0 |
| 69 | Rio Uruguai (RS, BRA/ CR, ARG) | -29,014 | -56,402 | 50 | 1496,50 | 565,80 | 0 | 7,23 | 1 | 0* | 0 |
| 70 | Rio Uruguai (RS, BRA/ CR, ARG) | -28,621 | -56,037 | 54 | 1435,60 | 624,80 | 0 | 7,22 | 1 | 0 | 1 |
| 71 | Rio Ibicuí (RS, BRA) | -29,415 | -56,753 | 47 | 562,00 | 498,90 | 0 | 6,80 | 1 | 0* | 0 |
| 72 | Rio Butuí (RS, BRA) | -28,932 | -56,272 | 53 | 100,60 | 585,70 | 0 | 5,49 | 1 | 0 | 0 |
| 73 | Icamaquã (RS, BRA) | -28,547 | -55,914 | 59 | 209,20 | 645,30 | 0 | 5,81 | 1 | 1 | 0 |
| 74 | Rio Uruguai (RS, BRA/ MI, ARG) | -27,847 | -55,025 | 92 | 1215,00 | 845,40 | 0 | 7,10 | 1 | 0* | 0 |
| 75 | Rio Uruguai (RS, BRA/ MI, ARG) | -27,735 | -54,914 | 92 | 1180,30 | 880,10 | 0 | 7,09 | 1 | 1 | 0 |
| 76 | Rio Uruguai (RS, BRA/ MI, ARG) | -27,852 | -55,030 | 96 | 1215,00 | 845,40 | 0 | 7,10 | 1 | 0* | 0 |
| 77 | Rio Uruguai (RS, BRA/ MI, ARG) | -27,916 | -55,256 | 85 | 1247,50 | 812,80 | 0 | 7,11 | 1 | 0* | 0 |
| 78 | Rio Uruguai (RS, BRA/ MI, ARG) | -27,895 | -55,130 | 90 | 1228,10 | 832,30 | 0 | 7,11 | 1 | 1 | 0 |
| 79 | Rio Tuparendi (RS, BRA) | -27,649 | -54,667 | 132 | 75,80 | 938,70 | 0 | 5,03 | 0 | 0 | 1 |
| 80 | Rio Uruguai (RS, BRA/ MI, ARG) | -27,565 | -54,674 | 108 | 1129,00 | 931,30 | 0 | 7,08 | 1 | 1 | 1 |
| 81 | Rio Santa Rosa (RS, BRA) | -27,569 | -54,627 | 124 | 126,80 | 932,30 | 0 | 5,26 | 0 | 0 | 1 |
| 82 | Rio Piratini (RS, BRA) | -28,119 | -55,396 | 73 | 247,10 | 787,60 | 0 | 5,87 | 1 | 0 | 0 |
| 83 | Rio Uruguai (RS, BRA/ CR, ARG) | -29,072 | -56,422 | 47 | 1502,50 | 557,90 | 0 | 7,23 | 1 | 1 | 0 |
| 84 | Rio Uruguai (RS, BRA/ CR, ARG) | -28,187 | -55,659 | 65 | 1321,20 | 739,20 | 0 | 7,18 | 1 | 1 | 0 |
| 85 | Rio Uruguai (RS, BRA/ CR, ARG) | -28,177 | -55,637 | 65 | 1320,80 | 739,60 | 0 | 7,18 | 1 | 1 | 0 |
| 86 | Rio Uruguai (RS, BRA/ MI, ARG) | -27,466 | -54,355 | 121 | 1062,80 | 997,60 | 0 | 7,06 | 1 | 1 | 0 |
| 87 | Rio Uruguai (RS, BRA/ CR, ARG) | -28,700 | -56,133 | 59 | 1443,60 | 616,80 | 0 | 7,22 | 1 | 1 | 0 |
| 88 | Rio Lajeado Grande (RS, BRA) | -27,403 | -54,210 | 139 | 3,40 | 1028,10 | 0 | 4,83 | 0 | 1 | 0 |
| 89 | Rio Lajeado Grande (RS, BRA) | -27,393 | -54,187 | 147 | 66,50 | 1028,10 | 0 | 4,83 | 0 | 1 | 0 |
| 90 | Rio Uruguai (RS, BRA/ MI, ARG) | -27,344 | -54,210 | 124 | 1030,30 | 1030,00 | 0 | 7,04 | 0 | 1 | 0 |
| 91 | Rio Buricá (RS, BRA) | -27,515 | -54,235 | 151 | 122,00 | 1027,90 | 0 | 5,47 | 0 | 1 | 0 |
| 92 | Río Miriñay (CR, ARG) | -28,947 | -57,275 | 65 | 9,60 | 544,60 | 0 | 5,23 | 0 | 0 | 0 |
| 93 | Río Aguapey (CR, ARG) | -27,825 | -56,258 | 78 | 94,40 | 719,40 | 0 | 5,33 | 0 | 0 | 0 |
| 94 | Río Gualeguaychú (ER, ARG) | -32,237 | -58,547 | 33 | 74,90 | 119,70 | 0 | 5,33 | 0 | 0 | 0 |
| 95 | Arroyo Tres Cruces (TA, URY) | -31,734 | -55,777 | 122 | 74,70 | 609,30 | 0 | 5,10 | 0 | 0 | 0 |
| 96 | Arroyo de las Canitas (TA, URY) | -31,680 | -56,447 | 182 | 19,00 | 473,90 | 0 | 4,13 | 0 | 0 | 0 |
| 97 | Río Tacuarembó (TA, URY) | -32,036 | -55,578 | 95 | 211,10 | 548,10 | 0 | 6,15 | 0 | 0 | 0 |
| 98 | Río Negro (TA, URY) | -32,623 | -55,827 | 79 | 419,60 | 357,00 | 0 | 6,68 | 0 | 0 | 0 |
| 99 | Río Negro (TA, URY) | -32,820 | -56,516 | 55 | 443,30 | 333,30 | 0 | 6,74 | 0 | 0 | 0 |
| N | Water bodies and localities | Latitude (W) | Longitude (S) | Elevation (m) | Upstream (km) | Downstream (km) | Yucumã (0) | Basin (km²) | *P. brachyura* | *P.  motoro* | *P.  sp.* |
|  |  |  |  |  |  |  |  |  |  |  |  |
| 100 | Rio Yi (FL, URY) | -33,476 | -56,161 | 88 | 143,20 | 319,40 | 0 | 5,91 | 0 | 0 | 0 |
| 101 | Rio Yi (FL, URY) | -33,263 | -56,813 | 54 | 231,60 | 231,00 | 0 | 6,21 | 0 | 0 | 0 |
| 102 | Rio Yi (FL, URY) | -33,373 | -56,566 | 67 | 195,00 | 267,50 | 0 | 6,09 | 0 | 0 | 0 |
| 103 | Arroio Itapitocai (RS, BRA) | -29,837 | -57,138 | 49 | 36,10 | 446,40 | 0 | 4,55 | 0 | 0 | 0 |
| 104 | Arroio Imbaá (RS, BRA) | -29,793 | -56,929 | 76 | 11,70 | 469,40 | 0 | 3,80 | 0 | 0 | 0 |
| 105 | Arroio do Salso (RS, BRA) | -29,798 | -57,092 | 56 | 17,70 | 442,10 | 0 | 3,71 | 0 | 0 | 0 |
| 106 | Río Negro (RV, UY) | -31,477 | -54,145 | 149 | 54,90 | 721,70 | 0 | 5,03 | 0 | 0 | 0 |
| 107 | Arroyo Cunapiru (RV, URY) | -30,905 | -55,558 | 191 | 14,20 | 745,00 | 0 | 4,11 | 0 | 0 | 0 |
| 108 | Arroio Carolina (RS, BRA) | -30,866 | -55,535 | 186 | 8,00 | 995,80 | 0 | 3,32 | 0 | 0 | 0 |
| 109 | Rio Quaraí (RS, BRA/ AR, URY) | -30,392 | -56,458 | 98 | 126,80 | 544,50 | 0 | 5,79 | 0 | 0 | 0 |
| 110 | Rio Pilão (RS, BRA) | -29,146 | -54,849 | 323 | 6,30 | 879,20 | 0 | 3,41 | 0 | 0 | 0 |
| 111 | Rio Ijuí (RS, BRA) | -28,324 | -54,257 | 207 | 171,10 | 1041,00 | 0 | 5,86 | 0 | 0 | 0 |
| 112 | Rio Ijuí (RS, BRA) | -28,163 | -54,774 | 148 | 294,00 | 918,10 | 0 | 6,10 | 0 | 0 | 0 |
| 113 | Rio Piratini (RS, BRA) | -28,460 | -55,130 | 123 | 172,40 | 862,50 | 0 | 5,72 | 0 | 0 | 0 |
| 114 | Rio Buricá (RS, BRA) | -27,675 | -54,164 | 177 | 86,60 | 1063,30 | 0 | 5,31 | 0 | 0 | 0 |
| 115 | Rio Santa Rosa (RS, BRA) | -27,771 | -54,359 | 206 | 23,50 | 998,40 | 0 | 4,19 | 0 | 0 | 0 |
| 116 | Rio Turvo (RS, BRA) | -27,388 | -53,883 | 215 | 130,50 | 1094,70 | 0 | 5,31 | 0 | 0 | 0 |
| 117 | Rio Conceição (RS, BRA) | -28,584 | -53,717 | 365 | 20,40 | 1140,90 | 0 | 4,27 | 0 | 0 | 0 |
| 118 | Rio Ijuí (RS, BRA) | -28,229 | -53,515 | 400 | 4,90 | 1171,00 | 0 | 4,75 | 0 | 0 | 0 |
| 119 | Rio Uruguai (RS, BRA/ MI, ARG) | -27,126 | -53,874 | 156 | 970,30 | 1090,10 | 1 | 7,01 | 0 | 0 | 0 |
| 120 | Rio Guarita (RS, BRA) | -27,322 | -53,653 | 227 | 125,50 | 1143,80 | 1 | 5,43 | 0 | 0 | 0 |
| 121 | Rio Guarita (RS, BRA) | -27,332 | -53,641 | 219 | 9,20 | 1145,40 | 1 | 5,43 | 0 | 0 | 0 |
| 122 | Rio da Várzea (RS, BRA) | -27,362 | -53,255 | 266 | 256,40 | 1224,10 | 1 | 5,84 | 0 | 0 | 0 |
| 123 | Rio Uruguai (SC, BRA/ MI, ARG) | -27,185 | -53,725 | 168 | 948,10 | 1112,20 | 1 | 6,99 | 0 | 0 | 0 |
| 124 | Rio Peperi-Guaçú (SC, BRA/ MI, ARG) | -26,430 | -53,687 | 488 | 29,40 | 1239,90 | 1 | 4,56 | 0 | 0 | 0 |
| 125 | Rio Peperi-Guaçú (SC, BRA/ MI, ARG) | -26,937 | -53,710 | 271 | 114,90 | 1154,40 | 1 | 5,43 | 0 | 0 | 0 |
| 126 | Rio Iracema (SC, BRA) | -27,064 | -53,290 | 239 | 53,40 | 1190,80 | 1 | 4,65 | 0 | 0 | 0 |
| 127 | Rio Uruguai (SC/ RS, BRA) | -27,134 | -53,405 | 295 | 888,30 | 1172,10 | 1 | 6,97 | 0 | 0 | 0 |
| 128 | Rio das Antas (SC, BRA) | -26,785 | -53,421 | 368 | 19,70 | 1227,80 | 1 | 2,67 | 0 | 0 | 0 |
| 129 | Rio Saudades (SC, BRA) | -26,829 | -53,040 | 395 | 29,00 | 1301,50 | 1 | 4,41 | 0 | 0 | 0 |
| 130 | Rio Chapecó (SC, BRA) | -26,918 | -52,915 | 270 | 293,70 | 1288,20 | 1 | 5,99 | 0 | 0 | 0 |
| 131 | Rio Uruguai (SC/RS, BRA) | -27,091 | -53,019 | 224 | 812,50 | 1247,80 | 1 | 6,85 | 0 | 0 | 0 |
| 132 | Rio da Várzea (RS, BRA) | -27,797 | -53,056 | 340 | 44,50 | 1329,00 | 1 | 5,53 | 0 | 0 | 0 |
| 133 | Rio Passo Fundo (RS, BRA) | -27,639 | -52,745 | 589 | 118,90 | 1391,50 | 1 | 5,41 | 0 | 0 | 0 |
| N | Water bodies and localities | Latitude (W) | Longitude (S) | Elevation (m) | Upstream (km) | Downstream (km) | Yucumã (0) | Basin (km²) | *P. brachyura* | *P.  motoro* | *P.  sp.* |
|  |  |  |  |  |  |  |  |  |  |  |  |
| 134 | Rio Erechim (RS, BRA) | -27,722 | -52,485 | 550 | 29,20 | 1420,20 | 1 | 4,79 | 0 | 0 | 0 |
| 135 | Rio Uruguai (SC/RS, BRA) | -27,249 | -52,591 | 259 | 715,70 | 1344,70 | 1 | 6,80 | 0 | 0 | 0 |
| 136 | Rio Chapecozinho (SC, BRA) | -26,797 | -52,591 | 391 | 158,10 | 1376,00 | 1 | 5,33 | 0 | 0 | 0 |
| 137 | Rio Chapecó (SC, BRA) | -26,865 | -52,756 | 342 | 25,80 | 1337,90 | 1 | 5,90 | 0 | 0 | 0 |
| 138 | Rio Chapecó (SC, BRA) | -26,605 | -52,490 | 608 | 18,90 | 1407,90 | 1 | 5,49 | 0 | 0 | 0 |
| 139 | Rio Xanxerê (SC, BRA) | -26,988 | -52,452 | 455 | 28,00 | 1401,40 | 1 | 4,35 | 0 | 0 | 0 |
| 140 | Rio Uruguai (SC/ RS, BRA) | -27,310 | -52,318 | 340 | 662,10 | 1398,30 | 1 | 6,76 | 0 | 0 | 0 |
| 141 | Rio Uruguai (SC/RS, BRA) | -27,377 | -51,989 | 351 | 590,90 | 1469,50 | 1 | 6,74 | 0 | 0 | 0 |
| 142 | Rio Jacutinga (SC, BRA) | -27,053 | -51,698 | 805 | 17,20 | 1504,70 | 1 | 4,18 | 0 | 0 | 0 |
| 143 | Rio do Engano (SC, BRA) | -27,074 | -52,136 | 572 | 36,00 | 1427,20 | 1 | 4,41 | 0 | 0 | 0 |
| 144 | Rio Uruguai (SC/ RS, BRA) | -27,512 | -51,795 | 406 | 522,80 | 1537,60 | 1 | 6,59 | 0 | 0 | 0 |
| 145 | Rio do Peixe (SC, BRA) | -27,417 | -51,771 | 426 | 222,80 | 1520,70 | 1 | 5,82 | 0 | 0 | 0 |
| 146 | Rio Forquilha (RS, BRA) | -27,713 | -51,749 | 565 | 115,00 | 1575,60 | 1 | 5,39 | 0 | 0 | 0 |
| 147 | Rio Apuê-Inhandava (RS, BRA) | -27,634 | -51,890 | 412 | 132,50 | 1550,10 | 1 | 5,59 | 0 | 0 | 0 |
| 148 | Rio do Peixe (SC, BRA) | -27,175 | -51,501 | 528 | 145,10 | 1598,40 | 1 | 5,70 | 0 | 0 | 0 |
| 149 | Rio Pelotas (SC, BRA) | -28,210 | -50,757 | 683 | 53,20 | 1754,50 | 1 | 6,05 | 0 | 0 | 0 |
| 150 | Rio São João (SC, BRA) | -27,392 | -51,158 | 884 | 22,50 | 1674,40 | 1 | 4,11 | 0 | 0 | 0 |
| 151 | Rio Canoas (SC, BRA) | -27,640 | -51,017 | 664 | 381,90 | 1678,40 | 1 | 6,24 | 0 | 0 | 0 |
| 152 | Rio Caveiras (SC, BRA) | -27,724 | -50,652 | 769 | 34,90 | 1761,30 | 1 | 5,35 | 0 | 0 | 0 |
| 153 | Rio Canoas (SC, BRA) | -27,866 | -51,066 | 553 | 332,40 | 1675,50 | 1 | 6,19 | 0 | 0 | 0 |
| 154 | Rio Canoas (SC, BRA) | -27,604 | -50,015 | 841 | 26,20 | 1917,80 | 1 | 5,55 | 0 | 0 | 0 |
| 155 | Rio dos Touros (RS, BRA) | -28,647 | -50,277 | 1005 | 28,80 | 1842,80 | 1 | 4,37 | 0 | 0 | 0 |
| 156 | Rio Cerquinha (RS, BRA) | -28,671 | -50,029 | 1183 | 5,30 | 1884,80 | 1 | 2,71 | 0 | 0 | 0 |

* Absence records excluded from modeling (absence records between presence records).
¹ Argentina (ARG): Corrientes (CR), Misiones (MI), Entre Ríos (ER); Brazil (BRA): Santa Catarina (SC), Rio Grande do Sul (RS); Uruguay (URY): Artigas (AR), Salto (SA), Durazno (DU), Flores (FL), Paysandú (PY), Río Negro (RN), Rivera. (RV), Soriano (SO), Tacuarembó (TA).
